# Comperative analysis of accuracy between low-frequency ultrasound biomicroscopy and 14-MHz ultrasonography with tissue harmonic imaging for the evaluation of the posterior lens capsule in traumatic cataracts

**DOI:** 10.1186/s12886-021-02094-z

**Published:** 2021-10-22

**Authors:** Bin Wu, Xiaoyong Yuan, Song Chen

**Affiliations:** Clinical College of Ophthalmology, Tianjin Medical University, Tianjin Eye Hospital, Tianjin Key Laboratory of Ophthalmology and Visual Science, Tianjin Eye Institute, Tianjin, China

**Keywords:** Ultrasound biomicroscopy, Ultrasonography, Traumatic cataracts, Posterior capsule

## Abstract

**Background:**

To compare the accuracy of low-frequency ultrasound biomicroscopy (LFUBM) and 14-MHz ultrasonography with tissue harmonic imaging (14-MHz + THI) in the assessment of posterior capsule (PC) integrity in patients with traumatic cataracts (TCs).

**Methods:**

From January 2019 to October 2020, 51 patients (51 eyes) with TCs who were scheduled for cataract extraction and for whom the PC of the lens could not be observed by the slit lamp visited Tianjin Eye Hospital, including 47 patients (47 eyes) with a penetrating injury of the eyeball and 4 patients (4 eyes) with a blunt injury of the eyeball. All eyes underwent LFUBM and 14-MHz + THI examinations before cataract extraction to determine the integrity of the PC. The integrity of the PC observed in surgery was the actual findings, and the consistency between the 2 methods was assessed in terms of the preoperative examination and intraoperative findings. Fisher’s exact test was used for consistency analysis, and *P* < 0.05 was considered statistically significant.

**Results:**

Thirty-two eyes with ruptured PCs and 19 eyes with intact PCs were actual findings in surgery. Thirty eyes with ruptured PCs and 21 eyes with intact PCs were examined by LFUBM. Thirty-two eyes with ruptured PCs and 19 eyes with intact PCs were examined by 14-MHz + THI. There were no significant differences between the 2 methods and the intraoperative findings *(P* = 0.293 LFUBM, *P* = 0.623 14-MHz + THI). The sensitivity, specificity, positive predictive value, negative predictive value and accuracy of LFUBM and 14-MHz + THI were 91 and 94%, 95 and 89%, 97 and 94%, 86 and 89% and 92 and 92%, respectively.

**Conclusions:**

Both LFUBM and 14-MHz + THI were proved to have high levels sensitivity and specificity in diagnosing the status of the PC in TC and they can be used as accurate diagnostic tool in these cases.

## Background

Blunt or penetrating ocular trauma is a recognized cause of cataract at any age [1]. The surgical management of traumatic cataract (TC) requires several considerations [2]. If the posterior capsule (PC) of the TC is ruptured, the operation cannot be carried out according to the conventional method. Depending on the degree of anterior segment disorder present, the location and size of PC rupture, and the degree of posterior segment tissue damage, a reasonable operation method and an appropriate intraocular lens should be implanted in a timely manner to avoid intraoperative and postoperative complications and to allow the injured eye to recover as soon as possible. Therefore, it is very important to determine the integrity of the PC before surgery.

In recent years, there have been many advancements in optical scanning methods, anterior segment optical coherence tomography (AS-OCT), Scheimpflug imaging and long-range sweep source optical coherence tomography (SS-OCT), and these methods can be used to display, quantify and analyze anterior eye tissue, especially the lens, to a certain extent. For example, the position of the foreign body in the lens and the integrity of the PC can be detected, the degree of lens opacity can be evaluated, and tiny opacity of the lens can be observed. These methods are convenient, fast, accurate and risk-free. However, with optical imaging systems, the equatorial part of the lens cannot be fully displayed due to occlusion of the pupil, and when ocular trauma, corneal edema, aqueous humor inflammation, severe blood opacity, and severe lens cortex opacity, the transparency of the refractive stroma is poor, it is difficult to observe the PC of the lens or tiny PC ruptures [3–13].

Recently, it has been found that 14-MHz ultrasonography with tissue harmonic imaging (14-MHz + THI) can accurately evaluate the integrity of the PC of TCs [14]. Ultrasound biomicroscopy (UBM) can clearly show the tissue of the anterior segment, but its high frequency limits its penetrability. The frequency of low-frequency UBM (LFUBM) is 25 MHz. The lower frequency increases the depth of detection, allowing the whole white lens to be visualized. This method may be used to evaluate the integrity of the PC of TCs.

In this study, LFUBM and 14-MHz + THI were used to observe the integrity of the PC of TCs before surgery, and the findings were compared with the intraoperative findings to determine the accuracy of the 2 methods in evaluating the integrity of the PC.

## Methods

### Subjects

From January 2019 to October 2020, 51 patients with TCs scheduled for cataract extraction visited Tianjin Eye Hospital. The inclusion criterion was as follows: the integrity of the PC could not be determined in the slit lamp examination. The exclusion criteria were as follows: the eyeball wound was not completely closed; there was also ocular surface inflammation or endophthalmitis; and there was also massive vitreous hemorrhage. Patients meeting any of the above three conditions were not included in this study. This study was approved by the ethics committee of the Tianjin Eye Hospital, and all procedures were performed in accordance with the Declaration of Helsinki.

### Methods

#### Preoperative examination

All patients underwent slit lamp microscopy, 10-MHz B-scan ultrasonography, ultrasound biomicroscopy (UBM), and computed tomography (CT) examination preoperatively.

One day before the operation, an operator independently completed the LFUBM examination, and another operator independently completed the 14-MHz + THI examination. The two operators kept the examination results confidential. In the same brightness environment, the 14-MHz + THI examination was performed first. A color Doppler ultrasound imaging device (LOGIQ 7, GE company, USA) with a 12 L probe (14-MHz, linear array scanning), focus distance of 1–2 cm, depth of 3 cm, and harmonic imaging mode were used. The lens was scanned in all directions in the two-dimensional ultrasound mode. Then the LFUBM (MD-320 W, MEDA, CHINA, customized product, transducer frequency of 25 MHz, transverse resolution ≤0.15 mm, longitudinal resolution ≤0.08 mm, penetration ≥11 mm) examination was conducted. Patients were scanned in a supine position using an immersion technique the same as conventional UBM. The probe was adjusted along each angle to inspect the PC of the whole lens. Imaging was particularly to show the presence of the PC.

#### Evaluation of PC

The integrity of the PC was evaluated according to the method described in reference [15]. An intact PC was noted when the echo of the PC of the equatorial posterior lens was continuous and smooth, showing a regular semilunar arc. PC rupture was noted when either 1. there is a middle echo passage in the lens that passed through the PC to the vitreous body or 2. the PC lost its original half-moon arc shape and was irregularly wavy with a discontinuous echo.

#### Intraoperative observation of the PC

According to the methods provided in reference [16], two experienced cataract surgeons observed the PC during the operation. If the cortex fell into the vitreous cavity or the vitreous body overflowed during the process of aspiration of the turbid cortex, a rupture of the PC was confirmed; after the cortex or nucleus around the turbid area was removed, the cortex and PC at the turbid area were separated gently with a viscoelastic agent, and the state of the PC was observed after careful aspiration of the cortex. The operation was performed gently to avoid iatrogenic PC rupture.

### Statistical methods

The same statistical methods presented in reference [3] were used. SPSS 17.0 software was used for statistical analysis. Intraoperative observation of the PC integrity was considered gold standard, and the findings were compared with those of LFUBM and 14-MHz + THI. Fisher’s exact test was used for consistency analysis, and *P* < 0.05 was considered statistically significant. The sensitivity, specificity, positive predictive value, negative predictive value and accuracy of the two methods were calculated. The sensitivity was calculated as the true positive/true positive + false negative. The specificity was calculated as the true negative/true negative + false positive. The positive predictive value was calculated as the true positive value/total positive value. The negative predictive value was calculated as the true negative/total negative. The accuracy was calculated as the true positive + true negative/total cases.

## Results

**Fig. 1:**
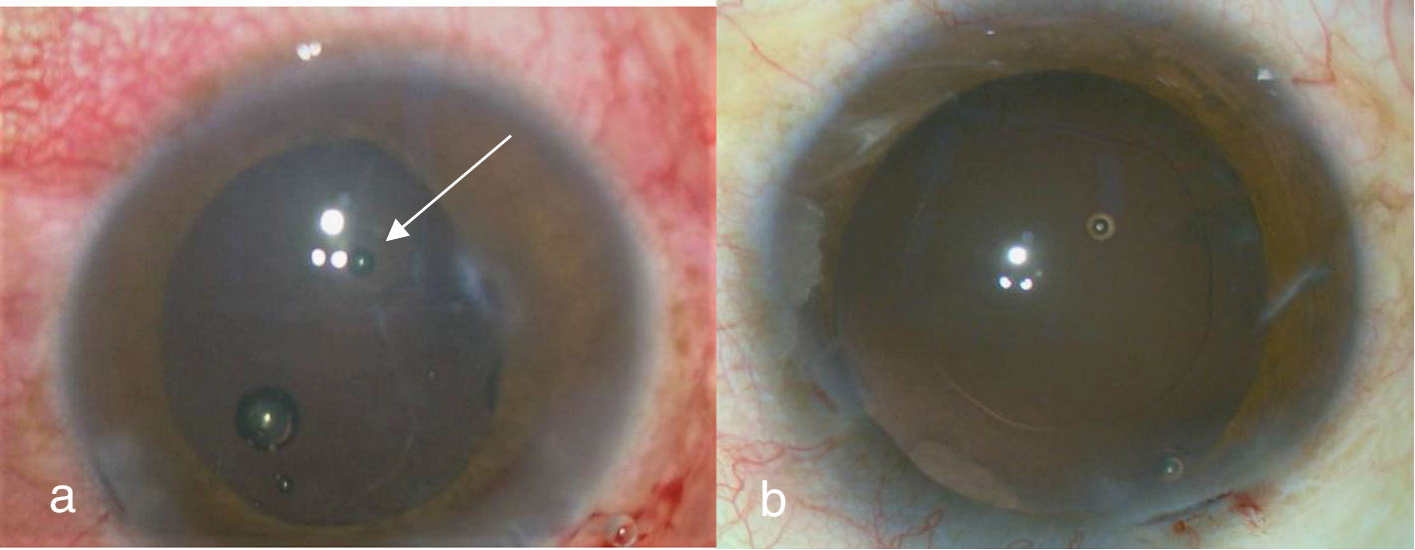
a The PC observed in surgery was ruptured (arrow). b: The PC observed in surgery was integrity

**Fig. 2:**
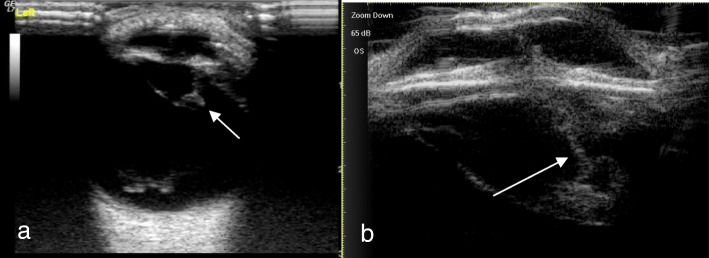
a The true positive image taken with 14-MHz + THI. The lens is hypoechoic and irregularly conical in shape, the PC is sharp, penetrating the vitreous body, and the rupture is moderately echoic (short arrow). b: The true positive LFUBM image. The ultrasonic performance is similar to that shown in a, with a medium echo penetrating channel in the crystal body (long arrow)

**Fig. 3:**
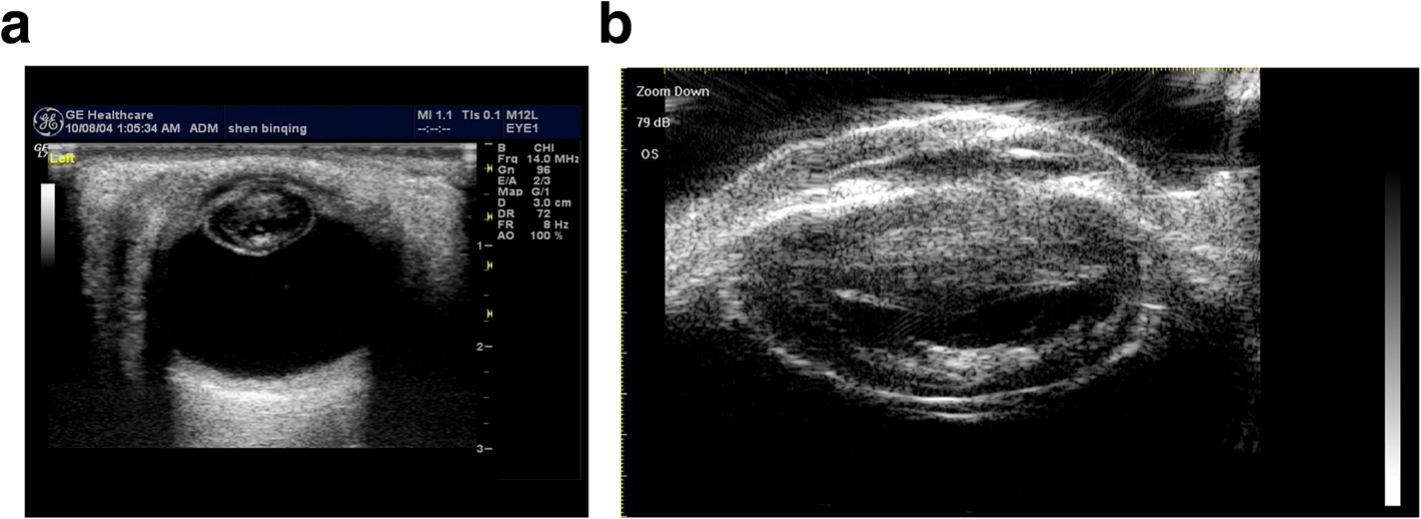
a The true negative image taken with 14-MHz + THI. The PC is smooth and round with a continuous echo. b: The true negative LFUBM image. The ultrasonic performance is similar to that shown in a

**Fig. 4:**
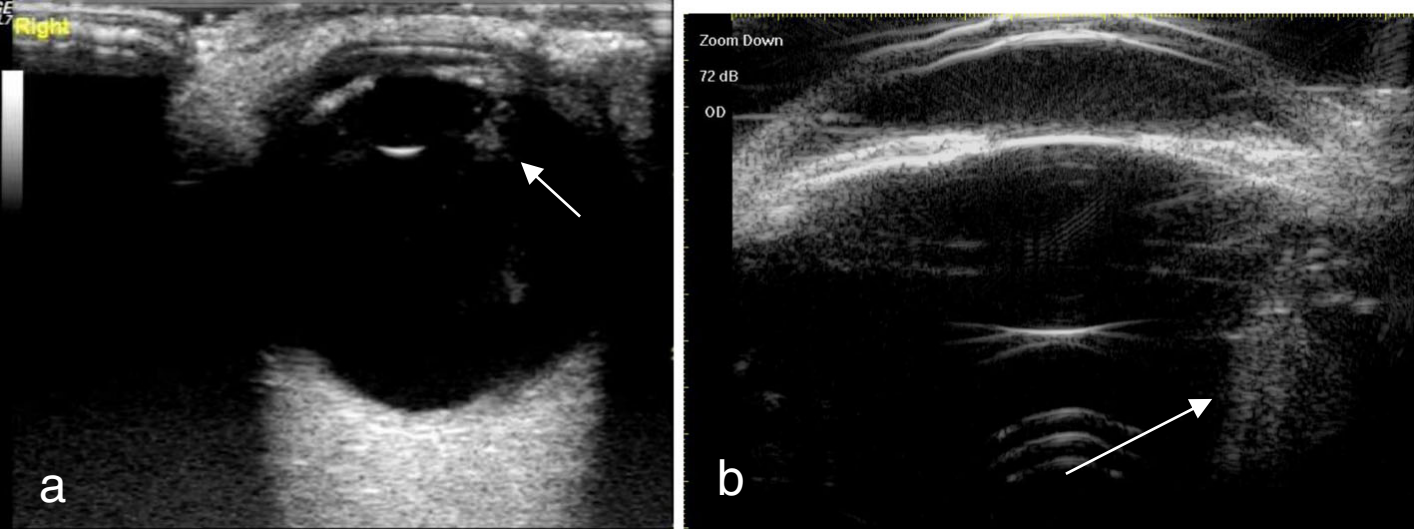
a The false positive image taken with 14-MHz + THI. A moderate echo mass is seen in the equatorial part of the lens, which is mistaken for a PC rupture and cortex overflow (short arrow). b: False positive LFUBM image. The ultrasonic performance is similar to that shown in a (long arrow)

**Fig. 5:**
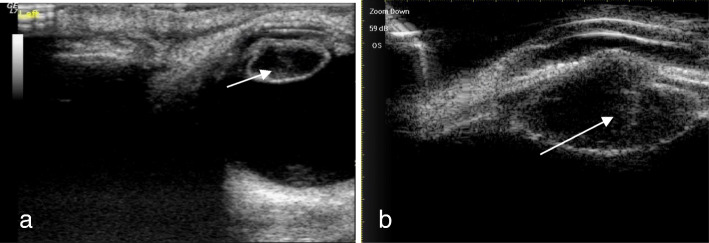
a The false negative image taken with 14-MHz + THI. The anterior capsule of the lens is ruptured, the echo of the PC in the lens is regular and continuous, passage is seen in the lens (short arrow), the PC is not penetrated, and the PC is mistaken for being intact. b: The false negative LFUBM image. The ultrasound performance is similar to that shown in a (long arrow)

**Fig. 6:**
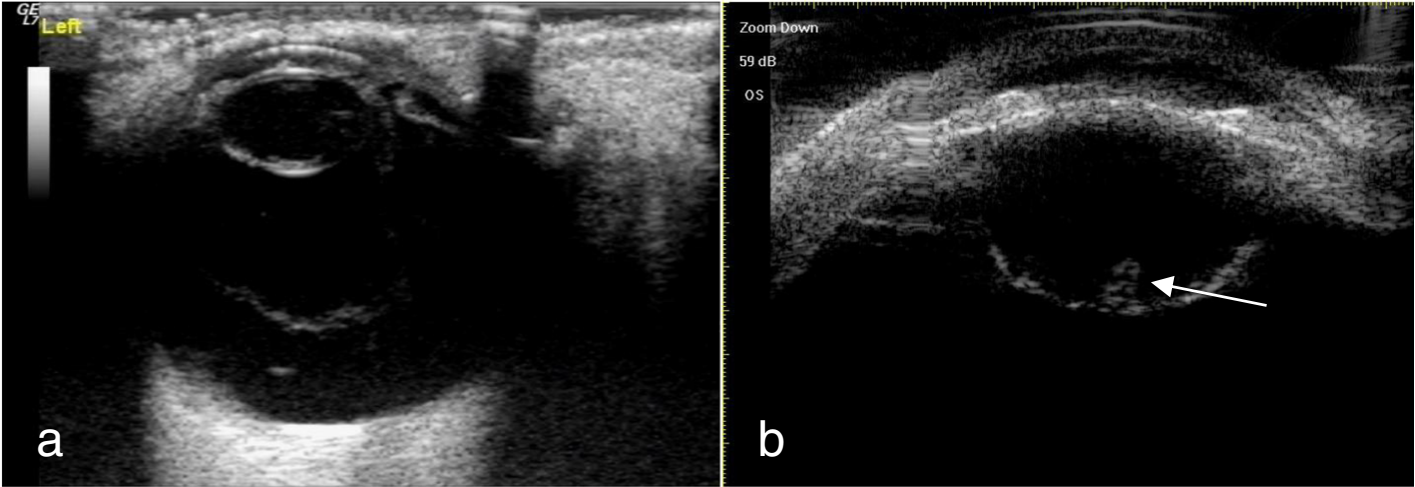
a The false negative image taken with 14-MHz + THI. Hypoechoic signals are present inside the lens, the anterior and posterior capsules are smooth, and the echo is homogeneous and complete. b: The true positive LFUBM image. The echo of the PC of the lens is smooth and continuous, and a moderate echo can be seen in front of the PC of the posterior pole, which is the rupture (arrow)

**Fig. 7:**
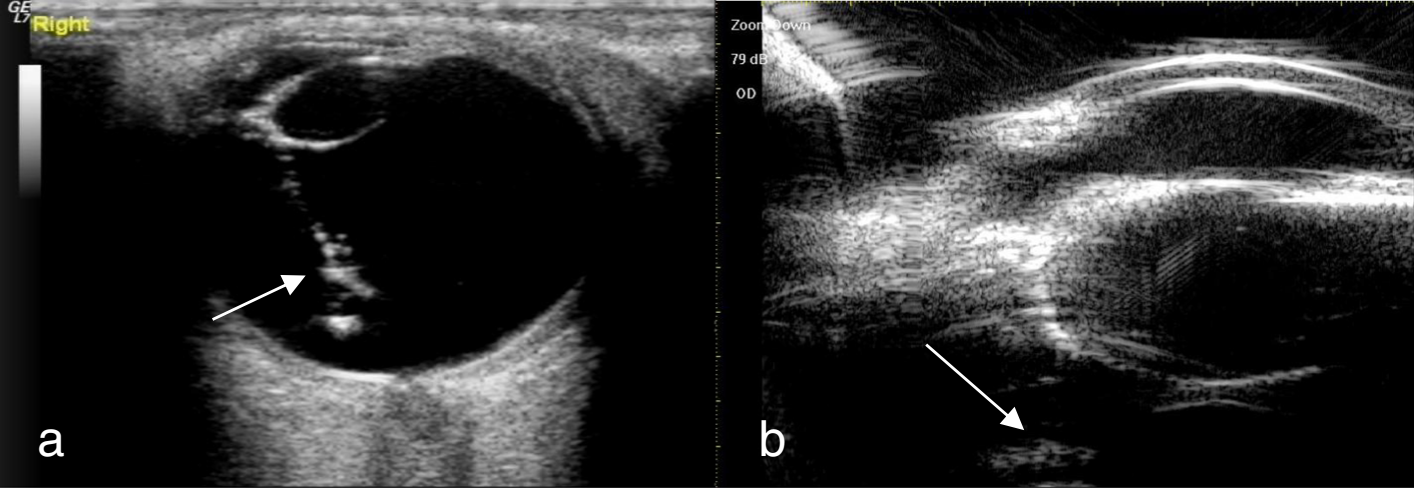
a The true positive image taken with 14-MHz + THI. The echo of the PC of the lens is regular and continuous, there is middle and high echo passages from the equator of the lens to the vitreous body, and the punctate middle and high echo in the vitreous body is the suspected lens cortex (short arrow). b The false negative LFUBM image. The echo of the PC of the lens is regular and continuous, and a moderate echo can be seen behind the equator, which is mistaken for vitreous opacity (long arrow)

A total of 51 eyes from 51 patients, including 38 males (38 eyes) and 13 females (13 eyes), with an average age of 35.17 ± 11.32 years (range, 17–68 years), were analyzed. There were 47 patients (47 eyes) with a penetrating injury and 4 patients (4 eyes) with a blunt injury. Table 1 shows Comparison of intraoperative findings with 2 modalities in state of PC. There were no significant differences in PC integrity between the intraoperative findings and each of the 2 imaging methods (*P* = 0.623 14-MHz + THI, *P* = 0.293 LFUBM). The PC rupture could be clearly observed in surgery (Fig. 1a and b).14-MHz + THI and LFUBM images of PC rupture showed a middle echo passage through the lens to the vitreous body. Some of the lenses were seriously deformed, and the PC was discontinuous (Figs. 2a,b,6b,7a). However, the intact PC images showed the PC echoes was continuous, and the anterior capsule of some lenses was ruptured (Fig. 3a,b). With14-MHz + THI, 2 eyes had a false positive (Fig. 4a), 2 eyes had a false negative (Figs. 5a and 6a), and all of these eyes had penetrating injuries. With LFUBM, 1 eye had a false positive (Fig. 4b), 3 eyes had a false negative (Figs. 5b and 7b), and all of these eyes had penetrating injuries. Table 2 shows the sensitivities, specificities, positive predictive values, and negative predictive values.

**Table 1:** Comparison of intraoperative findings with 2 modalities in state of PC

Parameter	Intraoperative findings	14-MHz + THI	LFUBM
Ruptured	32(31P + 1B)	32(31P + 1B)	30(29P + 1B)
Intact	19(16P + 3B)	19(16P + 3B)	21(18P + 3B)

*P* penetrating injury, *B* blunt injury

**Table 2:** Sensitivities, specificities, positive predictive values, negative predictive values and accuracy of the 2 modalities

Parameter	14-MHz + THI	LFUBM
Sensitivity	0.94	0.91
Specificity	0.89	0.95
Positive predictive value	0.94	0.97
Negative predictive value	0.89	0.86
Accuracy	0.92	0.92

## Discussion

Most TCs have complicated injury conditions and complicated morphological characteristics after lens injury, making the operation very difficult to perform and leading to many intraoperative and postoperative complications and poor prognoses [17]. If we can understand the overall shape of the lens before surgery, especially the integrity of the PC, we can select the appropriate surgical strategy and predict the prognosis. The results of this study show that there are no significant differences between the 2 imaging methods with respect to the intraoperative observation method in the assessment of PC integrity, and these methods can be used to assess PC integrity before surgery. In this study, there were three reasons for false positives: 1. There was an echo of vitreous opacity in the equatorial part of the lens, which was mistaken for overflow of the lens cortex and a rupture of the PC. 2. There were long and narrow perforating channels in the lens. The turbid cortical echo produced a tail shadow on the PC, which was mistaken for perforating channels through the PC. 3. The anterior capsule of the lens was severely ruptured, a large amount of lens cortex overflowed into the anterior chamber, the tension of the PC decreased, and the echo was wavy, which was mistaken for PC rupture. This study suggests that when TCs caused by a fine sharp object, the ruptures of the anterior and posterior capsules are small, the shape of the lens is still intact, and the PC still forms a semilunar arc, false negative results are more likely. Compared with 14-MHz + THI, LFUBM has a higher probe frequency, larger image and clearer display of the PC. However, LFUBM is affected by the depth of detection and cannot reveal the vitreous body. The frequency of UBM ranges from 50 MHz to 100 MHz. The higher frequency limits the depth of exploration (≤ 5 mm). The lens of some TCs expands, and UBM cannot display the whole picture of the lens [18, 19]. Decreasing the ultrasonic frequency of UBM can increase the depth of exploration. Kucukevcilioglu et al. [20] clearly observed a wound less than 1 mm in the PC of a lens with 35 MHz UBM. This study also showed that LFUBM can accurately evaluate the integrity of the PC. To observe the PC of the lens, 20 MHz B-scan is widely used. Study have shown that the accuracy of its evaluation is 88.37% [15]. The higher the ultrasound frequency is, the higher the resolution and the worse the penetration [21]. Based on this conception, the resolution of LFUBM is higher than that of 20 MHz B-scan. However, whether the accuracy of a small PC rupture is better than that of 20 MHz B-scan needs to be studied further. Whether 20 MHz B-scan or LFUBM, it is necessary to ensure that the eye wound is completely closed before the examination, and the instruments that are to come in contact with the eye need to be disinfected; otherwise, there is a risk of infection. No eye infections were found in any of the eyes in this study. Compared with 14-MHz + THI, 14-MHz + THI is more comfortable for patients and has no risk of infection, but its equipment cost is high, so it is not easy to popularize. LFUBM is inexpensive, lightweight, and portable. The examination is performed in the same way as conventional UBM examinations, so the equipment is easy to operate, but LFUBM has high requirements for patient compliance and is not suitable for some children and elderly people.

In this study, we found that LFUBM and 14-MHz + THI can accurately evaluate the integrity of PC of the lens, comprehensively assess the risk of surgery before surgery, and be used to develop targeted surgical plans to reduce the risk of surgery.
